# “Strong Teeth”: the acceptability of an early-phase feasibility trial of an oral health intervention delivered by dental teams to parents of young children

**DOI:** 10.1186/s12903-021-01444-z

**Published:** 2021-03-20

**Authors:** Amrit Bhatti, Kara A. Gray-Burrows, Erin Giles, Lucy Rutter, Jayne Purdy, Tim Zoltie, Robert M. West, Sue Pavitt, Zoe Marshman, Peter F. Day

**Affiliations:** 1 School of Dentistry, University of Leeds, Clarendon Way, Leeds, LS2 9JT UK; 2 University of Sheffield, Sheffield, UK; 3 Bradford Community Dental Service, Bradford District Care NHS Foundation Trust, Bradford, UK

**Keywords:** Caries, Training, Behaviour change, Paediatric, Prevention, Parents

## Abstract

**Background:**

Dental caries (tooth decay) in children is a worldwide public health problem. The leading cause of caries is poor oral hygiene behaviours and the frequent consumption of sugary foods and drinks. Changing oral health habits requires effective behaviour change conversations. The dental practice provides an opportunity for dental teams to explore with parents the oral health behaviours they undertake for their young children (0–5 years old). However, evidence suggests that dental teams need further support, training and resources. Therefore, “Strong Teeth” (an oral health intervention) was co-developed to help dental teams undertake these behaviour change conversations. The current paper will explore the acceptability of the “Strong Teeth” intervention with dental teams and parents of children aged 0–5 years old using multiple datasets (interviews, focus groups and dental team member diaries)

**Methods:**

Following the delivery of the “Strong Teeth” intervention, qualitative interviews with parents and focus groups with dental team members were undertaken. Interviews were audio-recorded, transcribed and analysed using a theoretical framework of acceptability. The self-reported dental team diaries supplemented the interviews and focus groups and were analysed using framework analysis.

**Results:**

Four themes were developed: (1) integration within the dental practice; (2) incorporating the Oral-B electric toothbrush; (3) facilitating discussions and demonstrations; and (4) the practicality of the Disney Magic Timer app. Overall, the “Strong Teeth” intervention was acceptable to parents and dental teams. Parents felt the Oral-B electric toothbrush was a good motivator; however, the Disney Magic Timer app received mixed feedback on how well it could be used effectively in the home setting. Findings suggest that the intervention was more acceptable as a “whole team approach” when all members of the dental practice willingly participated.

**Conclusions:**

There are limited studies that use a robust process evaluation to measure the acceptability of an intervention. The use of the theoretical framework of acceptability helped identify aspects of the intervention that were positive and helped identify the interventions areas for enhancement moving forwards. Future modifications include enhanced whole team approach training to optimise acceptability to all those involved.

***Trial registration*:**

ISRCTN Register, (ISRCTN10709150).

## Introduction

Dental caries (tooth decay) is the most prevalent childhood condition worldwide, affecting 2.4 billion people [1] and as such, reducing the prevalence of dental caries is a significant public health priority [2]. In England, the number of children affected by caries is substantial, however, within deprived areas, these numbers are significantly higher, with 17% of children aged 3 years experiencing caries, and 40% of 5-year-olds [3]. Children who have caries experience pain, loss of sleep, and problems with eating and speaking [4]. Furthermore, dental caries can affect the general health and quality of life in children, impacting their nutrition, school attendance and school performance [5].

Caries, however, is preventable [6]. Supporting parents to initiate and adopt protective home-based oral health behaviours in early-life is crucial to the development of long-term good oral health habits [6, 7]. Previous research, however, has identified that changing oral health behaviour is challenging, especially once dental disease has been established. As such, there has been an impetus to develop oral health interventions for young children to establish good oral health habits from the outset utilising existing workforces [8].

Dental teams are a key workforce providing preventative support to parents with young children; however, the effectiveness of one-to-one behaviour change conversations is limited [9]. Several studies have cited barriers to dental-led conversations, including sporadic opportunities for delivery, a lack of training, insufficient time, poor resources and a lack of consistency in how and what information is given [10–15]. Although the Public Health England’ Delivering Better Oral Health’ (DBOH) guidance identifies what advice to give, what is lacking is guidance on *how* to effectively undertake these behaviour change conversations to support parents to adopt good oral health habits for their children at home [16]. NICE [17] guidance states that conversations underpinned by behaviour change approaches ensure better outcomes. This highlights the need for effective support, training and resources to enhance the dental teams’ behaviour change skills, enabling them to support parents with young children effectively. Critically, interventions that attempt to empower dental teams in this way need to be evaluated to assess their effectiveness. The first step in this process are early-phase studies to explore acceptability, feasibility and potential impact. There are few published feasibility studies which undertake multiple qualitative methods or multiple datasets (triangulation) to explore the acceptability of interventions as part of their evaluation. By doing so, researchers can enhance data richness, and explore the phenomena further using additional qualitative data. As such, the current study will adopt a multi-construct Theoretical Framework of Acceptability [18] as a framework to assess the effectiveness and implementation of the intervention.

“Strong Teeth” (https://www.dentalcare.co.uk/en-gb/strong-teeth-strong-kids) is a complex oral health intervention, which is underpinned by appropriate psychological theory and a robust co-design methodology. The intervention development process is beyond the scope of the current paper and is reported in detail elsewhere [8, 14, 16, 19, 20]. In summary, “Strong Teeth” provides evidence-based resources to support oral health conversations between the dental team and parents, and training for dental teams on how to have an effective behaviour change conversation guided by the resources, an Oral-B electric toothbrush and an agreed delivery protocol. This paper focuses on the qualitative results from an early-phase feasibility study of the “Strong Teeth” intervention. A graphical summary of the “Strong Teeth” intervention procedures has been provided to clarify the sequence and timing of training, baseline data collection, intervention, follow-up data collection and interviews/focus groups (see Additional file 1). A protocol paper also provides a full description of the early-phase evaluation of the “Strong Teeth” intervention [19]. The quantitative results are reported in a separate paper [20].

## Aim

Using multiple datasets (interviews, focus groups and self-reported dental diaries) to explore the acceptability of the “Strong Teeth” intervention with dental teams and parents of children aged 0–5 years old.

## Research design and methods

### Parent sample

Parents (n = 27,) who completed the 2-month follow-up visit were offered a qualitative interview (see Additional file 2 for recruitment flowchart). Twenty parents agreed to be interviewed within their home setting (convenience sample). Reason for non-participation included work and other time commitments. Recruitment and retention to the study as well as quantitative findings are presented in detail in Giles et al. [20] paper with the summary flowchart provided in the additional files.

### Dental practice sample

Each dental practice that participated in the study was invited to participate in a focus group following the delivery of the final “Strong Teeth” intervention. Five focus groups were held with the dental practices within the West Yorkshire district. The focus group invitation was also extended to the wider dental team, including receptionists, managers, dental nurses, dentists, hygienists, and therapists. This enabled the research team to explore the suitability of the Strong Teeth intervention for uptake across the practice. This wider topic is beyond the scope of the paper and will be reported at a later date. In total, 22 dental team members were interviewed (convenience sample).

### Dental diaries

Following the delivery of the “Strong Teeth” intervention, 28 out of 34 structured diaries were completed by the dental team member and returned to the research team.

### Ethical approval

Ethical approval was obtained by the Health Research Authority (HRA) ID: 248833 and Health and Care Research Wales (HCRW). Ref: 18/YH/0326.

### Data collection

Method triangulation involves the use of multiple methods of data collection about the same phenomenon [21]. The types of triangulation used within the current study included (1) individual interviews, (2) focus groups, and (3) dental diaries.

#### Individual interviews and focus groups

For the interviews and focus groups, participants were aware that all information discussed would remain anonymous and confidential. All participants involved in the interviews/focus groups gave both verbal and written consent, and both followed a semi-structured interview guide. The interview guide covered a range of topics, such as the acceptability of the research process and acceptability of the intervention, thoughts on the resources and training, and suggestions for the improvement or development of the intervention for future implementation. The topic guide was based on a similar one used and tested in an earlier intervention study called HABIT [22]. After the interview/focus groups took place, the wider research team discussed whether there needed to be any modification to the interview guide in response to emerging findings. AB also wrote field notes after the interviews/focus groups to provide reflexivity and to create an audit trail [23].

#### Dental diaries

After delivering each “Strong Teeth” intervention, dental team members completed a semi-structured diary. This noted their thoughts on the intervention, what oral health barriers were identified within the appointment, and what “Strong Teeth” resources were used during the appointment (see Additional file 3). All data was anonymised, including the name of the parent and practice. The diaries were sent to a member of the research team (JP) and analysed at the end of data collection. The dental diaries were co-developed at the “Strong Teeth” training day, where dental teams had the opportunity to provide feedback or suggest changes to the document.

#### Parents

Interviews with parents were undertaken between February and August 2019 within the home setting. A member of the research team (AB) contacted the parents after the final round of data collection to arrange a convenient time and date for the interview. The interviews lasted between 20–50 min and were undertaken by one researcher (AB) who was already known by the parents from earlier data collection visits. These earlier visits helped to build rapport and familiarity with parents to facilitate an open discussion.

#### Dental team

The five focus groups with dental team members occurred between May and June 2019 with two facilitators (AB and JP). Focus groups took place in quiet rooms within the dental practice. All focus groups lasted approximately 1 h. Dental team members who took part in the intervention were sent information sheets at the beginning of the study and attended a training day, which provided the opportunity to meet the research team and ask questions. Dental team members who were not recruited for the “Strong Teeth” study were given verbal information about the focus group by their manager and a consent form, and information sheet was given on the day of the focus group. One of the researchers (JP) was known to the practice as she had regular contact with the dental team members throughout the study.

### Data analysis

All audio recordings of the interviews/focus groups were professionally transcribed. Data were anonymised and stored securely. Data were analysed using Framework Analysis [24]. Transcripts from all interviews were read by a member of the project team (AB). This was a part of the process of analysis (familiarisation with the data). Data were coded using the theoretical framework of acceptability (TFA) by Sekhon [18] within an excel spreadsheet. This consisted of seven components: affective attitude, burden, perceived effectiveness, ethicality, intervention coherence, opportunity costs, and self-efficacy (full details and description of these components have been presented in Table 1). In line with the framework method [24], the coded data were then summarised within the framework matrix, which enabled a large amount of data to be organised. The summaries within the matrix were then used to develop themes and sub-themes.

**Table 1:** The theoretical framework of acceptability with adapted definitions

Acceptability construct	Definition
Affective attitude	How parents/dental team members felt about the “Strong Teeth” intervention
Burden	The perceived amount of effort that is required to participate in the “Strong Teeth” intervention
Ethicality	The extent to which the “Strong Teeth” intervention has a good fit with parents/dental team members’ value system
Intervention coherence	The extent to which parents/dental team members understand the intervention and how it works
Opportunity costs	The extent to which benefits, profits or values must be given up engaging in the “Strong Teeth” intervention
Perceived effectiveness	The extent to which the “Strong Teeth” intervention is perceived as likely to achieve its purpose
Self-efficacy	The parents/dental team members’ confidence that they can perform the behaviours required (e.g., Deliver the intervention) to participate in the “Strong teeth” intervention

Adapted from: Acceptability of healthcare interventions: an overview of reviews and development of a theoretical framework [18]

The diaries supplemented the interviews and focus groups. The self-reported dental diaries were analysed at the end of data collection and after the analysis of the interviews and focus groups. Themes which were developed by the individual interviews and focus groups were put into a framework in excel [25]. Data was coded using the framework, and summaries within the matrix were used to develop themes and sub-themes.

The analysis across these data sets used an iterative, pragmatic approach, whereby the themes were developed and changed over time. At the end of the analysis, multiple researchers (PD, AB, KG-B, EG, JP and ZM) from different disciplines (dentistry and psychology) were involved in peer debriefing. This multi-method, multidisciplinary collaborative research was insightful. It enabled cross-validation and facilitated the exploration of issues that influenced the acceptability and feasibility of the intervention. All reached a consensus, aided investigator triangulation and ensured credibility and rigour until saturation had been reached.

#### “Strong Teeth” resources

Both parents and dental team members discuss the “Strong teeth” resources in depth during the focus group and interview discussions and as such, details of the these have been presented and the resources are also available online at https://www.dentalcare.co.uk/en-gb/strong-teeth-strong-kids#Resources, including, the tent card, conversational flowchart, leaflets and websites.

## Results

The lead author, while undertaking the analysis, identified that there were many cross-cutting themes for both parents and dental team members, and are thus presented and discussed in combination. The themes were as follows: (1) integration within the dental practice; (2) incorporating the Oral-B electric toothbrush; (3) facilitating discussions and demonstrations; and (4) the practicality of the Disney Magic Timer app. Themes and sub-themes are presented in Table 2.

**Table 2:** Themes and sub-themes across interviews/focus groups with dental team members and parents and dental self-reported diaries related to the theoretical framework of acceptability [18]

Theme	Sub-theme
1. Integration within the dental practice	1.1 “Strong Teeth” intervention viewed as a “friendly chat.”
	1.2 Providing a comfortable environment
	1.3 A need to involve the wider dental team
2. Incorporating the Oral-B Electric toothbrush	2.1 Toothbrushing impact on the wider family
	2.2 Transition of electric toothbrush difficulties
	2.3 Oral-B electric toothbrush makes brushing “fun.”
	2.4 Costing of toothbrush products
	2.5 The Oral-B electric toothbrush is “Easier to navigate.”
	2.6 The electric toothbrush is a “next step” from manual/battery toothbrushes
3. “Strong Teeth” facilitating discussions and demonstrations	3.1 Resources create conversations
	3.2 Toothbrushing demonstration should be “mandatory.”
	3.3 Passing on oral health information to the wider family
	3.4 Number of leaflets given varied
	3.5 Resources provide structure
	3.6 Resources provide “support.”
4. The practicality of the Disney Magic Timer app	4.1 App involves the siblings
	4.2 App impacts routine
	4.3 Internet access is needed to run the App
	4.4 The practicality of devices in bathrooms

### Theme one: integration within the dental practice

Dental team members described how the “Strong Teeth” intervention aligned with their current practice (and therefore, value system) of having effective oral health conversations with parents (ethicality). The dental practices had a robust preventative focus, and as such, dental team members valued how the “Strong Teeth” intervention enabled the oral health conversations to be delivered as a “friendly chat”. Previously, parents identified oral health conversations to be one-way where they were being told what to do, in a negative and authoritative manner [16, 26, 27]. Equally, dental team members felt as though they were “lecturing” parents. Interestingly, however, both groups reported how the “Strong Teeth” intervention enabled a two-way, friendly conversation between parent and dental team members:

What was helpful for me was that I went in thinking, ‘I need to come out with a list of things that they’re not allowed to have, and I was ready for a bit of a bashing [laughs]. Whereas actually, that wasn’t really what I came out with. It was more like treats are okay, […] but just be careful and think about when you’re giving them to them and make it work for your family rather than restrict [them]. - Rachel (parent)

Although the “Strong Teeth” intervention was delivered by members of the wider dental team, dentists within the wider practice (but not directly involved in the study) were concerned with how long it would take to deliver the intervention and how this fitted with the current fee structure. Timing barriers have been highlighted within previous studies [15, 28], especially for high-risk children. These factors make the provision of preventive dental care, such as delivering the “Strong Teeth” intervention, appear time-consuming. Within the self-reported diaries, dental team members reported appointments lasting between 10 and 30 min. Dentists identified the 1-day training event and their restricted appointment times as a barrier to them delivering the “Strong Teeth” intervention. Despite this initial concern, dentists could see the potential for delivery by the wider dental team:

It’s something that could definitely be rolled out and integrated, it’s just engaging dentists because they don’t have that long for appointments, so they need to know that it’s not going to eat into their time. – Jack (dental team member)

The findings within the current study suggest that the dentist can reinforce oral health messages within an appointment, but the “Strong Teeth” intervention itself can be delivered by the wider dental team, such as oral health educators, dental nurses and dental hygienists. As such, the wider dental team was viewed as the “most appropriate person” to deliver oral health advice:

R1: The advice is great, but it wouldn’t fit in the time that we have. [dentist]

I: Who would be the best person to deliver it then?

R1: Oh, [name of dental nurse], the oral health educators, there’s no doubt about that.- Bill (dental team member)

The “Strong Teeth” intervention appealed to the wider dental team, encouraging a “whole team approach” to delivering better oral health and provided the opportunity to optimise every contact with parents. Previous research demonstrates that a whole team approach is essential in reducing oral health inequalities [29] and requires the active engagement of all members of the dental team, consistent with the established policy guidelines of making every contact count (MECC) NHS Health Education England [30].

### Theme two: incorporating the Oral-B electric toothbrush

The Oral-B electric toothbrush received by children aged 3–5 years old within the “Strong Teeth” intervention was viewed as a good incentive. Despite the preferences for, and the advantages of, using electric toothbrushes, the cost of powered toothbrushes was viewed as a concern for dental team members who were hesitant to recommend Oral-B electric toothbrushes for their patients, especially within the more deprived areas [15, 16]. Parents who were involved in the “Strong Teeth” intervention, however, often reported buying an electric toothbrush for themselves and their other children after participating in the study:

I’d never had an electric toothbrush before, and I’d recently bought one for myself. I hadn’t known about this whole like doing a quarter of your mouth at a time [laugh] and doing like little circles and stuff. So I think I’d probably been doing it wrong all along. I think it’s quite good that I know that now and I try and pass that on to my daughter. – Jess (parent)

Habits, such as toothbrushing, are largely acquired through observational learning and modelling [31], and therefore if parents subsequently purchase an electric toothbrush for wider family members, children will be more likely to continue using their Oral-B electric toothbrushes.

Overall, there appeared to be a preference for the Oral-B electric toothbrush over the manual and was viewed as a “next step” from either a manual toothbrush or battery-operated toothbrush, especially as their children grew older:

Yeah, I do like it, I mean [name of second child] used to have like a battery-powered one and obviously the one that we got from the dentist is a charging one. He said the other day that the charging ones are better, cause the battery ones like lose power after a couple a’ days. So we are thinking about [name of second child], getting [name of second child] a charging one. – Lucy (parent)

Lucy described how she purchased the electric toothbrush because her dentist recommended it. The practicality of the device appeared to be superior to battery-operated toothbrushes and appeared to be a transition stage as their child becomes more motivated and independent (perceived effectiveness). Furthering this, dental team members and parents highlighted the benefits of using the Oral-B electric toothbrush, including cleaning the teeth more effectively and motivating the child:

Its got better plaque control, and obviously, they have a smaller head, easier to brush the back - Joe (dental team member)

I really like the size of the head. It’s easier for me to see where I’m putting it. Also, I feel like if you’re just moving it in small increments, it’s doing everywhere that it needs to do. It’s not too big and less damage. I don’t feel like I’m gonna jab the inside of her mouth or hurt her, ’cause it’s just small […] you can just move it and still clearly see what you’re doing. So I was really impressed with the size. - Monica (parent)

Participants highlighted how the Oral-B electric toothbrushes made toothbrushing “fun” for the child and appeared to motivate them to use their Oral-B electric toothbrush (perceived effectiveness):

There was some that were just like so excited to come like oh I’m getting the electric toothbrush OMG [“Oh My God”]. - Melissa (dental team member)

Toothbrushes were viewed as a reward or gift, and as such, children appeared to be excited about using their toothbrushes. The features of the Oral-B electric toothbrush were appealing to the child, including the timer, which some parents stated had ensured that the child brushed their teeth for the recommended length of time. Thus, receiving the Oral-B electric toothbrushes within the intervention may have potentially increased the likelihood of increasing the performance of toothbrushing behaviour.

Although toothbrushing appeared to be fun and motivating for the child, participants stated transitioning from a manual toothbrush to an electric toothbrush was challenging at first:

She did say, I think she’s getting more used to it now, but at first, it’s like she’d put it in, and she went “no it’s too tickly”, but I think the more she uses it, the more she’s getting used to it. – Alison (parent)

Although the vibrations of the Oral-B electric toothbrush were reported to be “tickly”, children appeared to accept the toothbrush once their parents had persisted. Other parents, however, stated that this resistance would result in them going back to their existing manual toothbrush. This may suggest that parents felt less confident using the Oral-B electric toothbrush when their child became resistant, therefore decreasing their self-efficacy. Further anticipatory support and guidance around how to best integrate an electric toothbrush into daily toothbrushing practice for children may help increase parents confidence with using the electric toothbrush.

### Theme three: facilitating discussions and demonstrations

The theme “facilitating discussions and demonstrations” showed the acceptability and usefulness of the “Strong Teeth” resources, beyond the dental setting. Overall, the “Strong Teeth” resources received positive feedback from participants who discussed how they facilitated oral health conversations, not only between parents and the wider dental team, but also between friends and family. Studies, including the current, have identified that wider family members may view sweets as “treats” [32, 33]. Participants stated how the resources provided evidence-based support and helped encourage conversations (affective attitude).

Parents described how the leaflets supported their conversations with the wider family:

The leaflet for the family just reinforces what I tell her dad and my parents in official text. I think that did encourage us. – Emma (parent)

[the leaflet] says you should do it twice a day. Cause that argument of well yeah I only want to do it once, it’s like, ‘well actually, the guidelines suggest…’. So it’s nice to have a bit of, for me, yeah a bit a’ back up… I can reinforce the message that I’m already saying. Cause people just think that I just, I’m saying it for no reason. So I feel like if its written down and published, if somebody paid to get it printed they might listen a bit more. - Lucy (parent)

This was especially useful when the child was frequently cared for by their wider family, such as grandparents and childminders. Some parents highlighted how they previously tried to approach family members about their child’s oral health, but were unsuccessful. The leaflets supported parents to have oral health conversations with wider friends and family, especially where they were previously hesitant to do so.

Research has highlighted that solely providing leaflets within the appointments received less positive outcomes and retention of oral health information [34]. However, dental teams highlighted how the “Strong Teeth” resources were used as a structured approach to undertaking oral health conversations. The leaflets were used to guide parents through the conversation, providing both verbal and written advice, which was tailored to the parent and their motivation:

Even if you were given the leaflets, it’s not gonna encourage you to read them. It’s gonna be something that you stuff in your bag while you’re trying to pick your kids up and that you probably don’t end up looking at. Whereas when she’s actually sat down, and she’s going through it with you, I think you’re more inclined to ask questions and understand more. - Tasha (parent)

The parent above highlighted the likelihood of leaflets being discarded if they were to be provided at the end of the visit. This was more likely given dental team members previously highlighted the number of leaflets currently within the practice (see dental team member Joe below). Interestingly, parents were encouraged to read the information because the dental team members guided them through the sections within the visit, providing a *structured* approach to how the conversation was led:

It gives a bit more of a structure as to how we deal with things and parents can get more involved, I think, to be honest. I really think they’ve been very, very good. First, when we first came to the training, I thought ‘oh I love more pamphlets’ you know. Loads more outside in the yards just thrown away, but when I looked at them, they’re excellent really. – Joe (dental team member)

The positioning of the tent card varied, sometimes being placed within the clinic rather than the reception area. However, the narrative highlights how the “Strong Teeth” resources could be utilised by the whole dental team, rather than just the dentist by placing key messages and resources throughout the practice. Parents are able to think about their oral health concerns while they are waiting to see their dentist, and the “Strong Teeth” resources can enable parents to identify their own questions as highlighted below:

It helps to sort of create a dialogue, getting them to ask questions.[referring to the tent card] - Melissa (dental team member)

As well as the “Strong Teeth” leaflets and tent cards, participants also highlighted the usefulness of the toothbrushing demonstration within their dental appointment. Parents were shown how to use their Oral-B electric and manual toothbrush using a model. One parent described how modelling the use of toothbrushes should be “mandatory” across all practices:

Once you start getting children, or even yourself, for it to be mandatory for someone to show you how to brush your teeth - Claire (parent)

The narratives highlight the importance of visual demonstrations to help both dental team members and parents identify problematic toothbrushing techniques, and the positioning of these resources are important to encourage oral health conversations. Parents stated how they recognised their toothbrushing techniques were wrong after they had seen the demonstration. A common mistake was for parents to use the electric toothbrush in the same way as a manual toothbrush and continue to brush the teeth side to side rather than gently moving the brush from one tooth to the next.

However, it was apparent that there was variability in the number of leaflets given within the “Strong Teeth” intervention (intervention coherence). Upon analysis of the self-reported dental diaries and focus groups, many dental team members gave more than two leaflets within their appointment. This meant that a wide range of topics were covered and could have led to ‘information overload’. One dental team member within their diary noted:

I felt that once I started the delivery, I possibly gave out too much information for one visit. When mum ticked several boxes on [the] oral care chat sheet, I wasn’t sure how to prioritise but with hindsight I could have asked her what her main concern was – (dental team member diary)

In turn, this made it difficult for parents to remember the context of the resources:

R I think there were 3 [leaflets]

I Do you know what they were about?

R One of ’em was definitely about foods and stuff; I can’t remember now it’s been a while. - Karen (parent)

Both the narratives and the diaries suggest that further training and support is needed to provide clarity that only one leaflet should be provided to prevent information overload and improve the remembrance of oral health conversations. Providing one leaflet is likely to maintain focus, limit the time of appointment and can be a prompt to bring parents back for a further appointment.

### Theme four: practicality of the Disney Magic Timer app

Within the theme of the practicality of the “Disney Magic Timer” app, sub-themes regarding the usefulness and difficulties of the Disney Magic Timer app were described, including how it made brushing “fun”, involved the siblings, how the App impacted routine, the need for internet access and the practicality of devices in bathrooms. As such, the “Disney Magic Timer” app recommended within the “Strong Teeth” intervention received varied responses regarding the acceptability for both parents and dental team members.

Some participants described how the App made toothbrushing fun for the child and served as an excellent way to motivate them to develop good oral health habits:

But then they like the App as well they *really* loved the App. The ones that hadn’t looked at it I opened it up and showed them, and they all seemed quite happy the kids were loving it - Kate (dental team member)

For other parents, however, the “Disney Magic Timer” app was viewed as burdensome and potentially negatively impacted on their child’s routine:

I didn’t find the App as useful for us. I can see why it could work for others. One, I’m quite strict on the devices, so we don’t generally allow their devices upstairs, and they definitely wouldn’t usually be allowed one in the bathroom […] Once she got the App, she wouldn’t do her teeth without the App, whereas she’d already been doing her teeth twice a day for her whole life [...] At one point we started to download it onto her iPad just purely so they could do her teeth, cause she wouldn’t do her teeth without it. But when I stopped using it, it was fine. - Monica (parent)

Downloading the “Disney Magic Timer” app conflicted with some parents views of allowing devices in the bathroom, and for others, the “Disney Magic Timer” app meant that it took longer for their child to brush their teeth, especially when a good routine had already been established:

But then as well every day when we got a sticker she wanted to go through all the stickers, so toothbrushing turned into a ten to fifteen minute job. - Sarah (parent)

Whilst studies have highlighted the benefits of mobile apps to motivate children with brushing [35, 36], the current study has highlighted that for those whose routine had already been established, incorporating the “Disney Magic Timer” app was viewed as burdensome. This is because incorporating the timer disrupted a good oral health routine, often making toothbrushing a more protracted process for the family. However, for those who struggled to brush their child’s teeth, the App was viewed as helpful and motivating for the child (affective attitude).

## Discussion

A summary of results from the “Strong Teeth” intervention reported using constructs from the Theoretical Framework of Acceptability (TFA) [18] can be found in Table 3. Overall, participants had positive feelings about the “Strong Teeth” intervention. In terms of affective attitude, parents and dental team members valued the “Strong Teeth” resources and Oral-B electric toothbrush and felt it integrated well within their family life and practice. For parents who struggled to brush their child’s teeth, the Oral B Magic Timer App also received positive feedback. Delivering the “Strong Teeth” intervention for the dentist was viewed as burdensome due to the perceived time within a routine appointment. However, all participants felt that the wider dental team members (such as oral health nurses, hygienists, and oral health educators) could deliver the intervention more suitably within their appointments. While for some parents, Oral B Magic Timer App was acceptable, for other parents, the App was viewed as burdensome given that toothbrushing took longer than anticipated. Therefore, the acceptability of the Oral B Magic Timer app varied. Ethicality was coded in all transcripts relating to the dental team members. Most coding occurred around how well the “Strong Teeth” intervention fits well with the dental teams’ current practice of delivering better oral health and how this aligns with their values of prevention.

**Table 3:** A summary of results from the “Strong Teeth” intervention reported using constructs from the Theoretical Framework of Acceptability (TFA) by Sekhon, Cartwright [18]

Acceptability construct	“Strong Teeth” findings related to the acceptability framework
Affective attitude	Parents and dental team members valued the resources The Disney Magic Timer app received positive feedback for parents who struggled to brush their child’s teeth
Burden	Delivering the full intervention for the dentist was viewed as burdensome due to timing restrictions The Disney Magic Timer app for some parents was viewed as burdensome when a good routine was previously established
Ethicality	Delivering the intervention fits well with the dental teams’ current practice of delivering better oral health
Intervention coherence	Variability in the number of leaflets given
Opportunity costs	N/A
Perceived effectiveness	Toothbrushing and use of the Oral-B electric toothbrush was viewed as being more effective compared to a manual
Self-efficacy	Transitioning to an electric toothbrush can be difficult, and some parents may result in going back to a manual if their child is resistant

Intervention coherence about the “Strong Teeth” intervention had some potential areas of improvement. For example, there was variability in the number of leaflets given by dental team members which suggest further refinement of training and support to ensure the intervention is given consistently and is more acceptable to parents receiving the intervention. In terms of opportunity cost, there was no charge to parents to receive the “Strong Teeth” intervention and therefore, was viewed as acceptable to participants. The anticipated and the experienced effectiveness of the “Strong Teeth” intervention, were generally positive for both parents and dental team members, especially toothbrushing and use of the Oral-B electric toothbrush compared to a manual. Self-efficacy was typically coded when parents spoke about transitioning to the Oral-B electric toothbrush. Some parents may result in going back to a manual if their child became resistant and could feel less confident using the Oral-B electric toothbrush.

Aspects of the “Strong Teeth” intervention could be modified in order to raise acceptability from the findings of the current paper. In particular, the “Strong Teeth” intervention should have a particular focus on a whole team approach. Many studies across the health care sector highlight the benefits of teamwork [29, 30, 37], and the recent adoption of the ‘direct access’ arrangements in 2013, has enabled the wider dental team (such as dental hygienists, dental therapists and dental nurses) to undertake a range of preventive tasks [38]. A recent study, [29] concludes “mechanisms that support understanding of the different professional roles, enhance team communication, and develop practical processes that facilitate DCP contribution within a practice would benefit teamwork of all kinds.” [29], p460]. Future refinement would provide further support to committed teamwork within the dental practice and further enhance team communication and approach.

For example, the “Strong Teeth” tent card could be positioned within the reception area to provide optimum opportunities for the wider team (such as receptionists) to initiate oral health conversations while the parent waits for their dental appointment. This could allow the parent to think about their oral health concerns before they enter the surgery. The dentist could then reinforce the appropriate critical oral health messages tailored to the concern. A further appointment could be made with the wider dental team to enable a consistent, yet more in-depth conversation with the parent regarding the topic area using the “Strong Teeth” leaflets.

### Future training

The findings within the study have also identified some key fidelity issues in that dental team members need further support in understanding the intervention and how it works (intervention coherence). This is because there was a range in the number of leaflets given within the appointment. As such, the current paper identified contextual factors that influenced the delivery of the intervention. Some dental team members provided up to four leaflets within the appointment, which in turn meant that parents did not remember the context because so many topics were discussed. This is similar to other studies, which demonstrated a number of oral health messages could be viewed as ‘information overload’ [39].

Further training and support are needed to provide clarity that a maximum of one leaflet should be provided to prevent information overload and allow the behaviour change to be manageable. Training would include how to have an effective behaviour change conversation using behaviour change techniques that will emphasise the importance of using the correct resource [40, 41]. Furthermore, given the value of observing the correct use of the electric toothbrush, dental team members could benefit from how to incorporate this into their routine appointment. Having a framework for oral health conversations, further top-up training and immediate feedback with the dental diaries could ensure that the intervention is acceptable to deliver, as well as feasible. These supportive follow up sessions could be delivered remotely in line with studies such support oral health advice by telephone (REF).

### Strengths and limitations

In line with established approaches to intervention evaluation, we aimed to explore the acceptability and feasibility of “Strong Teeth” using multiple qualitative methods. Intervention studies have often been criticised for being developed without having sufficient knowledge of how the target population will receive the intervention activities [42]. Furthermore, there are few published studies which undertake multiple qualitative methods (triangulation) to examine the acceptability and feasibility of interventions within dentistry. Triangulation within this study can enable the researchers to identify any issues identified during one data set and explore the phenomena further using additional qualitative data [21]. This, in turn, enriches the evaluation as it offers a variety of datasets to explain differing aspects of the intervention.

Researchers have commented on the increased validity of study findings through triangulation and the collection of data using multiple methods [21]. A strength of this method of data collection is the opportunity to triangulate the data and to perform member checking [21]. The researchers were able to explore the self-reported diaries and populate the diary data according to the themes presented by the interviews. Triangulation was used within the current study to promote a more comprehensive understanding of the “Strong Teeth” intervention and to enhance the rigour the study [43].

Although regarded as a means to add richness and depth to a research inquiry, there are some concerns regarding the use of triangulation in research. Some authors state that triangulation assumes that the data from different research methods are comparable. In particular, each data set being of equal weight in the research inquiry [43–46]. In light of these concerns and recommendations [44, 47], we have acknowledged this, and taken the following steps: The data of dental team members and parents were analysed separately, then, the similarities between the interviews and self-reported diaries were identified. Overall, each data collection tool was appropriate for their purpose, and the self-reported dental diaries were used as a method of facilitating and supporting the findings of individual interviews and focus groups.

The findings within the current paper reached both redundancy and consistency. However, the generalisability of the study should be cautioned in terms of the sample. Dental team members were recruited because of their reputation for their strong preventive ethos. Thus, the experiences of these may differ from “regular” practices. Lastly, given that the parents and dental team members were recruited from the “Strong Teeth” intervention and were a convivence sample, it could be argued that the experiences of those who took part in the interviews may differ to sample would differ from those who would be randomly selected. The generalisability of the study should be, therefore, be cautioned.

## Conclusion

The use of the theoretical framework of acceptability part of a process evaluation to examine parents’ and dental team members acceptability of “Strong Teeth” was helpful in identifying aspects of the intervention that required modification, as well as the positive and negative features. Overall, the “Strong Teeth” intervention was acceptable to parents and dental teams. Further refinements are needed to maximise the impact and efficiency of the “Strong Teeth” intervention, including enhanced training to ensure a whole team approach. The Sekhon, Cartwright [18] framework has provided a robust structure to examine the acceptability of the “Strong Teeth” intervention.

## Supplementary Information

**Additional file 1:** A graphical summary of the “Strong Teeth” intervention procedures.

**Additional file 2:** Research process participant flowchart.

**Additional file 3:** Dental team member diaries.

## Data Availability

The datasets used during the current study are available within the additional files. Further information is also available from the corresponding author on reasonable request.
