# Supplementary material for: “Strong Teeth”: the acceptability of an early-phase feasibility trial of an oral health intervention delivered by dental teams to parents of young children
Source: BMC Oral Health. 2021 Mar 20;21:138. doi: 10.1186/s12903-021-01444-z (PMC7980542; doi:10.1186/s12903-021-01444-z)
Supplement: Supplementary file 1 — Additional file 1: A graphical summary of the “Strong Teeth” intervention procedures. [file 12903_2021_1444_MOESM1_ESM.docx]

Additional file 1: A graphical summary of the “Strong Teeth” intervention procedures

| Timeline | Parents | Dental teams |
| --- | --- | --- |
| “Strong Teeth” intervention training |  | a, b, c, d, e, f, g, h |
| Baseline | I, j, k, l, m |  |
| “Strong Teeth” intervention |  | n, o, p, q, r, s, t, u |
| Two-week follow up | v, l, m |  |
| Two-month follow up | v, l, m |  |
| Focus groups/ interviews | w | x |

| a | “Strong Teeth” training (full day): Consent for dental team members to take part in the study |
| --- | --- |
| b | “Strong Teeth” training (full day): Communications for behaviour change training/ rolling with resistance |
| c | “Strong Teeth” training (full day): training of the “Strong Teeth” resources (read through of resources, discussion of how to implement, role play using the resources and behaviour change, explanation of how to use resources) |
| d | “Strong Teeth” training (full day): How to instruct parents on using the Oral B electric toothbrush |
| e | “Strong Teeth” training (full day): Informing the team about study design |
| f | “Strong Teeth” training (full day): Explanation of dental diaries and how best to implement the dental diaries |
| g | “Strong Teeth” training (full day): How to identify potential participants and introduce the study |
| h | Potential participants who were interested were sent an information sheet by the dental teams. The dental practice released participant contact information to the team liaison |
| i | The research team contacted parents to arrange baseline data collection |
| j | Consent of research study and verbal information about the design of study alongside information sheet |
| k | A questionnaire including parent and child demographics, self-reported brushing habits, toothbrushing attitudes, dietary data and frequency and bedtime routines |
| l | Dental examination- BASCD charting system, Gingival inflammation and Oral Hygiene index |
| m | Parent-child (dyad) toothbrushing interaction recording |
| n | “Strong Teeth” intervention: Allow parents to complete tent card |
| o | “Strong Teeth” intervention: Assess parents motivation and follow conversation flowchart. If unmotivated, refer to motivation poster |
| p | “Strong Teeth” intervention: identify one concern highlighted by parent and use appropriate leaflet to support conversation |
| q | “Strong Teeth” intervention: Undertake oral health conversation |
| r | “Strong Teeth” intervention: Give appropriate toothbrush and toothpaste |
| s | “Strong Teeth” intervention: allow parents to develop an action plan |
| t | “Strong Teeth” intervention: Give leaflet to parent with an action plan |
| u | Complete dental diary: Questions on how the visit went, what oral health barriers were identified within the appointment, and what “Strong Teeth” resources were used during the appointment |
| v | Follow up- Questionnaire including parent and child demographics, self-reported brushing habits, toothbrushing attitudes, dietary data and frequency and bedtime routines, attitudes of intervention and Oral-B electric toothbrush. |
| w | Interviews: followed using interview guide A |
| x | Focus groups: followed using interview guide B |
