# Supplementary material for: “Strong Teeth”: the acceptability of an early-phase feasibility trial of an oral health intervention delivered by dental teams to parents of young children
Source: BMC Oral Health. 2021 Mar 20;21:138. doi: 10.1186/s12903-021-01444-z (PMC7980542; doi:10.1186/s12903-021-01444-z)
Supplement: Supplementary file 2 — Additional file 2: Research process participant flowchart. [file 12903_2021_1444_MOESM2_ESM.docx]

Additional file 2- Research process participant flowchart

**Figure 1: Participant flowchart, as outlined by CONSORT (Eldridge et al., 2016)**

**Invitations per Practice (P)**

**P1**= 22; **P2** = 18; **P3** = 24; **P4** = 36; **P5** = 26

**Invitations**

(n= 126)

**Dropped out prior to consenting** (n=11)

Reason: Unable to contact (n=4)

Changed mind about participating (n=6)

Unable to accommodate home visit (n=1)

**Participants accepted**

(n=47)

**Consent & Baseline**

(n=36)

**Consented per Practice**

**P1**= 8; **P2** = 8; **P3** = 5; **P4** = 6; **P5** = 9


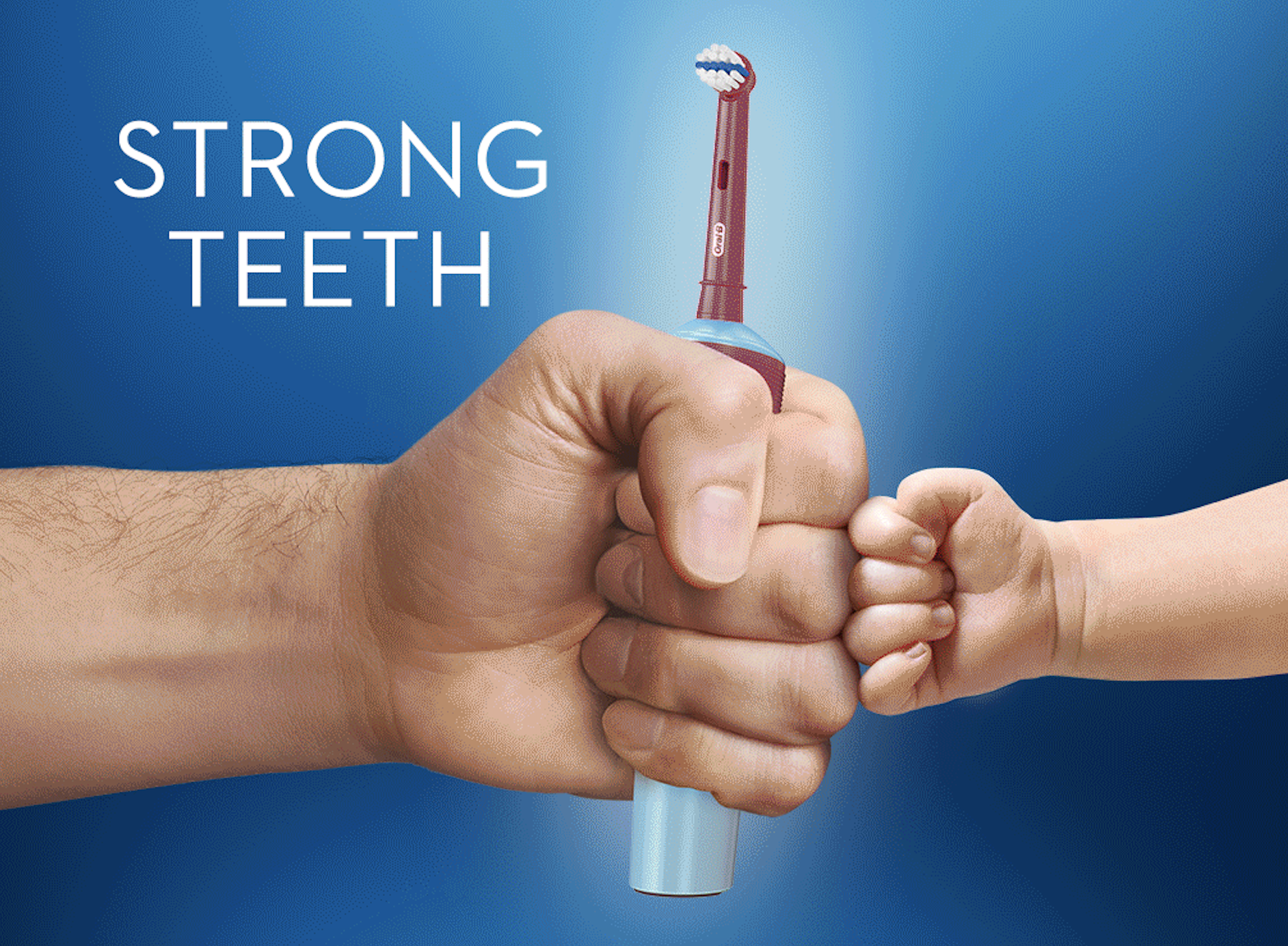


**Dropped out after baseline** (n=6)

Reason: Unable to contact (n=4)

Didn’t attend intervention appointment (n=2)

**Strong Teeth Invention n=34**

**2 Week follow up**

Completed: 26/30

**Missed follow up** (n=4)

Reason: Child Illness (n=2)

Away on holiday (n=1)

Delay in being able to contact (n=1)

**Dropped out after 2 week follow up** (n=3)

Reason: Unable to contact (n=3)

**3 Month follow up**

Completed: 27/27

**Missed follow up** (n=0)

**Declined participation** (n=6)

**Qualitative interviews**

Completed: 20/21

**Unable to interview** (n=1)

Reason: Participant work commitments (n=1)
