# Supplementary material for: “Strong Teeth”: the acceptability of an early-phase feasibility trial of an oral health intervention delivered by dental teams to parents of young children
Source: BMC Oral Health. 2021 Mar 20;21:138. doi: 10.1186/s12903-021-01444-z (PMC7980542; doi:10.1186/s12903-021-01444-z)
Supplement: Supplementary file 3 — Additional file 3: Dental team member diaries. [file 12903_2021_1444_MOESM3_ESM.docx]

Additional file 3: Dental team member diaries


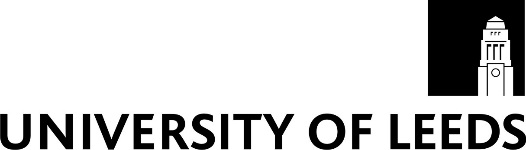


Outline of the Dental Team Member diary

**“Strong Teeth” programme: A study to explore the acceptability and impact of an oral health education programme delivered by dental teams to parents of young children**

Study Number: 248833, Version: 1, Date: 17-07-18

**Information about parent/child to whom the “Strong Teeth” programme oral health education has been provided:**

Date of the clinic visit: ………………………………………………………………………………………………………….

Who did you deliver the information to (e.g. mum, dad, other family member, friend, carer?) …………………………………………………………………………………………………………………………………

How many times have you delivered the “Strong Teeth” programme oral health education? (family number 1,2,3,4,5etc.)? …………………………………………………………………………………………….

**Generic information about the dental visit:**

Did the parent initiate discussion about oral health? Yes/ No

What were the oral health topics most important to the parent? ………………………………………….

…………………………………………………………………………………………………………………………………….………………………………………………………………………………………………………………………………………………………….

**Information about the “Strong Teeth” programme resources used to provide oral health education.**

Which barriers to good oral health did you focus on in this visit?

1. Motivation
2. Brushing for Ages 0-5
3. Tips for Getting Children to Brush
4. Healthy Eating
5. Friends and Family
6. Remembering to Brush

What was most important for the parent when talking about these barriers? ……………………….

………………………………………………………………………………………………………………………………………………..

……………………………………………………………………………………………………………………………….…………………………….……………………………………………………………………………………………………………………………………………….………………………………………………………………………………………………………………..

What resources did you provide the parent with and what was the parent’s reaction to the “Strong Teeth” programme resources? …………………………………………………………………………………

……………………………………………………………………………………………………………………………………………….……………………………………………………………………………………………………………………………………………….

Was the parent able to understand the “Strong Teeth” programme resources? Yes/ No

If yes, which components did they engage with most?

………………………………………………………………………………………………………………………………………………

……………………………………………………………………………………………………………………………………………….

Did the parent disagree with any part/ subject of the “Strong Teeth” programme resources? …………………………………………………………………………………………………………………………….

………………………………………………………………………………………………………………………………………………………………………………………………………………………………………………………………………………………………

How long did it take to deliver the “Strong Teeth” programme?

………………………………………………………………………………………………………………………………………………

Do you feel a further visit is needed to support parents to adopt good oral health behaviours? Yes/ No

If a further visit is needed – what would you like to concentrate on at this visit?

..…………………………………………………………………………………………………………………………………………..

**Information about the resources used to provide oral health education:**

How did you use the “Strong Teeth” resources during the appointment? ……………………………..

............................................................................................................................................................................................................................................................................................................................................................................................................................................................

Did you feel comfortable using the resources? ……………………………………………………………………..

………………………………………………………………………………………………………………………………………………………………………………………………………………………………………………………………………………………………

What did you like most about using the resources? ………………………………………………………………

……………………………………………………………………………………………………………………………………………….

……………………………………………………………………………………………………………………………………………….

What did you like least about using the resources? ……………………………………………………………….

……………………………………………………………………………………………………………………………………………….

……………………………………………………………………………………………………………………………………………….

What would you change about the resources or how they are delivered? …………………………….

...........................................................................………………………………………………………………………

……………………………………………………………………………………………………………………………………………….……………………………………………………………………………………………………………………………………………….

**For children aged between 3-5 years old**

Did you give out an Oral –B electric toothbrush? Yes/ No

If yes, would you recommend it over a manual brush [Yes/No]?

If no, what would be the reason(s)?...........................................................................

……………………………………………………………………………………………………………………………

If yes, what was the reaction of the child and parent to the electric toothbrush?

.......................................................................…………………………………………………………………………

………………………………………………………………………………………………………………………………………………………………………………………………………………………………………………………………………………………………

Did you manage to explain to the parent how to use the toothbrush? …………………………………

.......................................................................…………………………………………………………………………

………………………………………………………………………………………………………………………………………………………………………………………………………………………………………………………………………………………………

Do you have any other comments about the electric toothbrush? ……………………………………….

......................................................................…………………………………………………………………………

……………………………………………………………………………………………………………………………………………………………………………………………………………………………………………………………………………………………...
